# The standardization of the Polish version of the Alberta Infant Motor Scale

**DOI:** 10.1186/s12887-023-04055-5

**Published:** 2023-05-12

**Authors:** Małgorzata Eliks, Sowińska Anna, Steinborn Barbara, Ewa Gajewska

**Affiliations:** 1 Chair and Clinic of the Developmental Neurology, Poznan University of Medical Sciences, Poznań. Poland, Przybyszewskiego Street 49, Poznan, 60-355 Poland; 2 Doctoral School, Poznan University of Medical Sciences, Poznań. Poland, Bukowska Street 70, Poznan, 60-812 Poland; 3 Department of Computer Science and Statistics, Poznan University of Medical Sciences, Rokietnicka Street 7, 60-806 Poznan, Poland

**Keywords:** Alberta Infant Motor Scale, Infancy, Motor development, Standardization, Normative values

## Abstract

**Background:**

The Alberta Infant Motor Scale (AIMS) is a standardized tool for assessing gross motor development from birth through independent walking (0–18 months). The AIMS was developed, validated and standardized in the Canadian population. Results of previous studies on the standardization of the AIMS have discerned differences in some samples in comparison with Canadian norms. This study aimed to establish reference values of the AIMS for the Polish population and compare them to Canadian norms.

**Methods:**

The research involved 431 infants (219 girls, 212 boys, aged 0-<19 months), divided into nineteen age groups. The translated into Polish and validated version of the AIMS was used. The mean AIMS total scores and percentiles for every age group were calculated and compared with the Canadian reference values. Raw total AIMS scores were converted to 5th, 10th, 25th, 50th, 75th, and 90th percentiles. A one sample t-test was used to compare the AIMS total scores between Polish and Canadian infants (p-value < 0.05). A binomial test was performed to compare percentiles (p-value < 0.05).

**Results:**

The mean AIMS total scores in the Polish population were significantly lower in the seven age groups: 0-<1, 1-<2, 4-<5, 5-<6, 6-<7, 13-<14, and 15-<16 months of age (with small to large effect size). A few significant differences were found in the comparison of percentile ranks, mostly in the 75th percentile.

**Conclusion:**

Our study provides the norms for the Polish AIMS version. According to differences in the mean AIMS total scores and percentiles, the original Canadian reference values are not congruent for Polish infants.

**Trial registration:**

ClinicalTrials.gov ID NCT05264064. URL https://clinicaltrials.gov/ct2/show/NCT05264064. Date of registration: 03/03/2022.

## Background

The Alberta Infant Motor Scale (AIMS) is a standardized tool for assessing gross motor development from birth through independent walking (0–18 months). The AIMS was developed, validated and standardized in the early 1990s by Martha C. Piper and Johanna Darrah from the University of Alberta, Canada [1, 2].

The AIMS was created to monitor the motor skills achievements of infants with typical motor development and those at risk of developmental concerns [1, 2]. So far, the tool has been used as an outcome measure in studies on infants, e.g. born preterm [3, 4], with structural brain disorders [5–8], after surgical treatment of congenital cardiac defects [9, 10], affected by genetic diseases [11–13], or nonsynostotic plagiocephaly [14, 15].

The AIMS assessment relies on the observation of the spontaneous motor performance of an infant [1]. Besides minimal handling of an infant, other advantages comprise a relatively short duration of examination and ease of administration [16].

According to recommendations, the implementation of developmental assessment tools in populations other than the original should be preceded by cultural adaptation and validation in populations and languages other than initially considered [17, 18]. Previously, the scale has been validated in populations such as Taiwanese, Japanese, Brazilian, Spanish, Thai, Serbian, Korean, and Polish [19–28]. The results of the psychometric values of these versions were good or excellent.

Results of previous studies on the standardization of the AIMS have discerned differences in some samples in comparison to the Canadian norms. The research on Flemish and Dutch populations found significantly lower overall mean AIMS total scores in these samples [29–31]. In the Thai population, lower scores were noted in the first three months, whereas infants aged 7-<8 months, 11-<12 months, and 13-<14 months had considerably higher scores relative to the Canadian norms [16]. The results of the research in the Brazilian context are diverse. Gontijo et al. noted the majority of differences in the mean AIMS total scores relative to the Canadian reference values in the first six months of age (mainly lower scores) [32]. On the contrary, Saccani et al. found overall lower scores in the Brazilian sample [33]. The AIMS scores in Greek and Turkish infants were the most consistent with the original Canadian norms [34, 35]. The significantly lower scores were noted in Turkish infants aged 0-<1 and 1-<2 months of age [34], while in the Greek sample, a higher score was found only in the group of 2-<3 month of age [35]. The variability in the AIMS scores between populations indicates a need to standardize the tool across ethnic and cultural contexts before using it in clinics or research.

This paper is a part of the project on the introducing the Polish version of the AIMS. The first step included cultural adaptation and validation of the Polish AIMS scoresheet [21]. Now we aim to establish reference values of the AIMS for the Polish population and compare them to the Canadian norms. The standardization is needed to use the Polish version of AIMS in further research and clinical practise.

## Methods

### Participants

The study involved 431 infants between 5 days and 18 months 29 days divided into nineteen age groups with 1-month intervals. The inclusion criteria were (1) a gestational age between 37 and 42 weeks and (2) a birth weight of ≥ 2500 g, (3) a 5-min Apgar score ≥ 8. In turn, the exclusion criteria comprised (1) a gestational age < 37 weeks, (2) a birth weight < 2500 g, (3) a 5-min Apgar score < 8, and (4) any neurological, orthopedic, genetic, metabolic, and sensory disorders. Every infant underwent routine pediatric appointments according to the standard of Polish medical healthcare system. The recruitment was carried out *via* targeted advertisements on parenting-related websites, antenatal classes, nurseries, and neonatal and pediatric outpatient departments in the Greater Poland region. Parents or caregivers were asked to fill out a questionnaire on the infant’s condition. All parents or caregivers expressed their written consent to their children’s participation in the study. The research was conducted in agreement with the Declaration of Helsinki and the Bioethics Committee of Poznan University of Medical Sciences University (approval no. 1034/19).

## Instrument

### Alberta Infant Motor Scale

The validated Polish version of the AIMS was implemented [21]. The AIMS scoresheet consists of 58 items at four positions (21 in prone, 9 in supine, 12 in sitting, and 16 in standing) [1]. The evaluation of every item includes three components: weight-bearing, posture, and antigravity movements [1]. A drawing of the infant’s position accompanies every item [1]. An infant is assessed while the observation of spontaneous movement with minimal handling, e.g. encouragement with using a toy [1]. An examiner is to identify the least and the most mature items in every position – these constitute the developmental “window” and then to score every item in the “window” as “observed” or “not observed” [1]. Each item below the least mature is treated as “observed”. The scoring is dichotomous for each item – “observed” (1 point) or “not observed” (0 points) [1]. The sum of all the items (maximum of 58 points) in every position composes the total raw score, which may be converted into percentile ranks (with 1-month age group intervals) [1]. The assessment lasts 20–30 min. The examination methodology was concordant with the recommendation of the authors of the AIMS [1]. A fully fed and well-rested infant wearing a diaper was placed on a rehabilitation table or mat in a warm room during the assessment. The assessments were performed by two examiners – physiotherapists specialized in pediatric physiotherapy experienced in developmental assessments, and early intervention. Data were collected between 2020 and 2022. The study was carried out in the Chair and Clinic of the Developmental Neurology, Poznan University of Medical Sciences.

### Data analysis

The minimal sample size was determined as 380 participants (alpha = 0.05, error rate = 5%) based on the data from Statistics Poland.gov on number of births in Greater Poland region with criteria such as a gestational age > 37 weeks and birth weight < 2500 g (data of the year of 2018). The minimal size of the age group was set at 20 participants. Descriptive statistics are presented as a percentage for categorical variables, as mean with standard deviation for continuous variables. Raw total AIMS scores were converted to 5th, 10th, 25th, 50th, 75th, and 90th percentiles. A one sample t-test was used to compare the AIMS total scores between Polish and Canadian infants (p-value < 0.05 was considered as significant). A binomial test was performed to compare percentiles (p-value < 0.05 was considered as significant). Dell Inc. software was used for calculations (2016) Dell Statistica (data analysis software system), version 13. software.dell.com. The effect size was defined by calculating Cohen’s (d) with recognized benchmarks as small (0.2), moderate (0.5), and large (0.8) [36].

## Results

All participants were analyzed together on account of no significant differences between infants born before 39 weeks and at/after 39 weeks of pregnancy. The characteristics of participants is listed in Table 1.

Table 2 presents the mean and the standard deviation of the AIMS total scores in Polish and Canadian infants, and the minimum and maximum AIMS scoring in the Polish sample in every age range.

In Polish infants, the maximal score was noted for the first time in the group of 11-<12 months (in 8% of participants). Then it was achieved by 25% of individuals in the group of 13-<14 months, 84% of participants in the group of 14-<15 months, and all infants older than 17 months. In analyzing ranges between the minimum and maximum AIMS scores, the biggest diversities were shown in age groups of 7-<8 months (range of 25 points), 8-<9 months (range of 19 points ), 9-<10 months (range of 23 points).

The mean AIMS total scores in the Polish population were significantly lower than the Canadian values in the groups of 0-<1, 1-<2, 4-<5, 5-<6, 6-<7, 13-<14, and 15-<16 months of age. Small to large effect size was noted, with the majority of moderate.

**Fig. 1:**
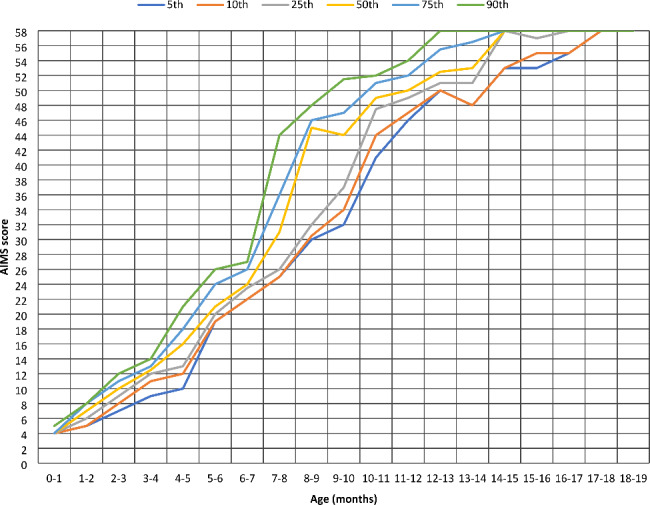
Percentile ranks of the AIMS score in Polish infants

Table 3 presents the percentile ranks in the Polish AIMS scores and the comparison with the Canadian norms. The significant differences were found in the 5th percentile in 3-<4, 15-<16, 16-<17 months of age, in the 10th percentiles in 15-<16 and 16-<17 months of age, in the 25th percentile in 6-<7 and 15-<16 months of age, in the 50th percentile in 6-<7 and 13-<14 months of age, in the 75th percentile in 0-<1, 1-<2, 3-<4, 5-<6, 6-<7, 11-<12 months of age, in the 90th percentile in 14-<15, 15-<16, 16-<17 months of age. Figure 1 shows the percentile of the AIMS score curves for Polish infants.

**Table 1:** Characteristics of participants

		n (%)	mean (SD)	median	min-max
**Sex**	female	219			
	male	212			
**birth weight (g)**			3478.43 (443.13)	3460	2500–4850
	8	1			
**5-min Apgar score**	9	21			
	10	409			
**gestational age (wk)**	37-<38	17			
	38-<39	65			
	39-<40	117			
	40-<41	121			
	41-<42	93			
	42	18			
**birth method**	natural	303			
	cesarean section	128			
**birth order**	1	271			
	2	139			
	3	15			
	4	4			
	5	2			

**Table 2:** The mean AIMS total score of Polish and Canadian infants for age groups

Age (months)	Polish infants						Canadian infants				
	n	mean	SD	min	max		n	mean	SD	**p-value**	**Cohen’s d**
0-<1	21	4.24	0.44	4	5		22	4.5	1.37	0.012*	0.26
1-<2	27	6.63	1.31	4	9		56	7.3	1.96	0.013*	0.40
2-<3	29	9.79	1.61	7	13		118	9.8	2.42	0.572	0.00
3-<4	26	12.35	1.47	8	15		90	12.6	3.29	0.386	0.10
4-<5	25	15.92	3.51	9	24		122	17.9	4.15	0.009*	0.52
5-<6	24	21.88	2.82	16	27		189	23.2	4.75	0.031*	0.34
6-<7	24	24.46	2.72	16	31		225	28.3	5.5	< 0.0001*	0.89
7-<8	22	32.05	7.29	22	47		222	32.3	6.85	0.872	0.04
8-<9	20	40.80	7.18	30	49		220	39.8	8.69	0.541	0.13
9-<10	20	42.80	6.77	30	53		189	45.5	7.47	0.091	0.38
10-<11	20	48.75	3.67	40	57		155	49.3	5.92	0.511	0.11
11-<12	24	50.75	3.12	46	58		155	51.3	7.11	0.397	0.10
12-<13	20	53.40	2.80	50	58		124	54.6	4.52	0.07	0.32
13-<14	20	53.00	3.71	48	58		86	55.6	5.01	0.005*	0.59
14-<15	25	57.24	1.83	52	58		61	56.9	1.97	0.363	0.18
15-<16	23	57.13	1.69	53	58		40	57.8	0.45	0.02*	0.75
16-<17	20	57.55	1.00	55	58		49	57.8	0.55	0.277	0.31
17-<18	20	58.00	0.00	58	58		49	57.9	0.35	**-**	-
18-<19	21	58.00	0.00	58	58		30	57.7	0.64	**-**	**-**

n–number of participants, SD – standard deviation, min – minimum, max – maximum

*significant results with p < 0.05. Cohen’s d –effect size values for estimating effect size

**Table 3:** The comparison of the AIMS percentile in Polish and Canadian infants

Age (months)	5th Pol	5th Can	p-value	10th Pol	10th Can	p-value	25th Pol	25th Can	p-value	50th Pol	50th Can	p-value	75th Pol	75th Can	p-value	90th Pol	90th Can	p-value
0-<1	4	2.2	0.288	4	2.7	0.128	4	3.6	0.108	4	4.5	0.124	4	5.4	0.014*	5	6.3	0.128
1-<2	5	4.1	0.615	5	4.8	0.775	6	6	0.534	7	7.3	0.091	8	8.6	0.045*	8	9.8	0.122
2-<3	7	5.8	0.262	8	6.7	0.145	9	8.2	0.135	10	9.8	0.681	11	11.4	0.352	12	12.9	0.201
3-<4	9	7.2	0.035*	11	8.4	0.107	12	10.4	0.085	12.5	12.6	0.537	13	14.8	0.024*	14	16.8	0.098
4-<5	10	11.1	0.556	12	12.6	0.298	13	15.1	0.109	16	17.9	0.051	18	20.7	0.074	21	23.2	0.189
5-<6	19	15.4	0.386	19	17.1	0.385	20	20	0.975	21	23.2	0.095	24	26.4	0.031*	26	29.3	0.121
6-<7	22	19.3	0.645	22	21.2	0.813	23.5	24.6	0.005*	24	28.3	0.0001*	26	32	0.006*	27	35.4	0.104
7-<8	25	21	0.762	25	23.5	0.760	26	27.7	0.792	31	32.3	0.901	36	36.9	0.977	44	41.1	0.915
8-<9	30	25.5	0.499	30.5	28.7	0.431	32	33.9	0.415	45	39.8	0.635	46	45.7	0.980	48	50.9	0.771
9-<10	32	33.2	0.592	34	35.9	0.453	37	40.5	0.257	44	45.5	0.187	47	50.5	0.222	51.5	55.1	0.340
10-<11	41	39.6	0.375	44	41.7	0.289	47.5	45.3	0.452	49	49.3	0.616	51	53.3	0.156	52	56.9	0.202
11-<12	46	39.6	0.265	47	42.2	0.117	49	46.5	0.076	50	51.3	0.524	52	56.1	0.024*	54	58	0.148
12-<13	50	47.2	0.463	50	48.8	0.476	51	51.6	0.923	52.5	54.6	0.168	55.5	57.6	0.068	58	58	0.476
13-<14	48	47.2	0.777	48	49.2	0.494	51	52.2	0.140	53	55.6	0.037*	56.5	58	0.117	58	58	0.882
14-<15	53	53.7	0.629	53	54.4	0.559	58	55.6	0.519	58	56.9	0.535	58	58	0.401	58	58	0.007*
15-<16	53	57.1	< 0.0001*	55	57.2	< 0.0002*	57	57.5	0.007*	58	57.8	0.249	58	58	0.647	58	58	0.041*
16-<17	55	56.9	0.013*	55	57.1	0.022*	58	57.4	0.119	58	57.8	0.456	58	58	0.519	58	58	0.022*
17-<18	58	57.3	-	58	57.5	-	58	57.7	-	58	57.9	-	58	58	-	58	58	-
18-<19	58	56.6	-	58	56.9	-	58	57.3	-	58	57.7	-	58	58	-	58	58	-

Pol – the Polish values, Can – the Canadian values, *significant results with p < 0.05

## Discussion

The main purpose of our study was to provide the reference norms of the AIMS (mean scores and percentiles) in Polish infants and compare them with the original Canadian normative values. This study is the second in the world (following the study of Saccani et al.), which developed normative values in population other than Canadian by using translated and validated AIMS scoresheet.

We examined the group of 431 term infants at the age of 0-<19 months. The size of samples in previous studies varied from 270 to 2202 participants. Some of them included only full-term infants (Greek, Brazilian – the study of Gontijo et al., Thai, Turkish), the others also comprised those born preterm (Canadian, Flemish, Brazilian – the study of Saccani et al.) [1, 16, 30, 32–35]. Besides Polish and Canadian studies, only two research (de Kegel et al. on the Flemish population and Saccani et al. on the Brazilian population) included all age groups (0-<19) in their analyses [1, 30, 33]. Syrengelas et al. (the Greek sample), Kepenek-Varol (the Turkish sample) et al., and Gontijo et al. (the Brazilian sample) involved participants younger than 18 months of age, and Tupsila et al. (the Thai sample) younger than 14 months of age [16, 33–35]. Some of the authors explained it by the fact that infants are used to achieving the maximum of the AIMS scores before 18 months of age.

The Polish mean AIMS total scores were similar to or lower than the original Canadian values in most of age groups. The significantly lower scores were found in the first two months, between 4-<7 months and in the samples of 13-<14 and 15-<16 months (in seven age groups). This finding indicates that the Canadian norms are not appropriate for Polish infants. Previous studies also comprised references to the Canadian values. The scores in Greek and Turkish populations were the most consistent with the Canadian reference norms, significant differences were found only in one age group (2-<3) or in two groups (0-<1, 2-<3), respectively [34, 35]. In the Thai sample, significantly lower scores than the Canadian norms were noted in the first three months (0-<4), whereas higher scores were observed in three age groups: 7-<8, 11-<12, 13-<14 months of age [16]. The findings of studies on Brazilian infants are ambiguous. The study of Gontijo et al. showed differences only in five age groups – significantly higher scores in 0-<1, and lower in 1-<2, 4-<5, 5-<6, 10-<11 months of age [32]. Whilst the research of Saccani et al. showed significantly lower scores in fifteen age groups (0-<13, 14-<16, 18-<19) [33]. These results might be explained by methodological differences between studies in the selection of participants (full-term versus full-term and born preterm) and sample size (660 versus 1455).

Furthermore, we noted a ceiling effect of the AIMS scoring. In Polish infants the maximal score was noted for the first time in the group of 11-<12 months (in the 8% of participants). Then it was achieved by 25% of individuals in the group of 13-<14 months, by 84% of participants in the group of 14-<15 months, and by all infants older than 17 months. The stabilization of the scoring was shown by the age of 15 months. In the study on the Flemish sample, there was reported that 90% of participants scored the maximum of the AIMS at the age of 16 months and older [30]. In the Brazilian sample (study by Saccani et al.) stabilization of the AIMS score was noted by 16 months of age, in the Canadian population by the age of 15 months [1, 33, 37]. The maximum AIMS score was achieved by 7.3% of Thai infants at the age of 10-<11 and 75.6% of participants in the group of 13-<14 [16]. This result indicates that using the AIMS is limited to the age of achieving a skill of independent walking, which usually emerges considerably earlier than at 18-<19 months of age. On the other hand, the ability to walk alone (57th item of the AIMS) is considered by the World Health Organization (WHO) to be achieved by 18 months of age [38].

In analyzing percentile ranks, the curves of the 5th and 10th, as well as the 75th and 90th percentile overlap in the group of 1-<2 month. It can be explained by the relatively small variability of the range of AIMS scores at this age. The overlapping of the curves of the 25th, 50th, 75th and 90th percentiles starts at 14-<15 months of age, and this point refers to achieving the maximum AIMS score. Saccani et al. noted starting of the overlapping of the 75th to 99th percentile at 12 months. Darrah et al. observed the convergence of the 75th and 90th percentiles at 13 months of age [33, 37]. The curves of the 5th, 10th and 90th percentiles overlap in 17-<18 months of age. We consider as clinically meaningful that infants of the 5th and 10th percentiles achieved the maximum AIMS score by 18 months of age. The curves of the 5th and 10th percentiles are anigh almost along and convergent between groups of 5-<9 and 12-<19 months of age. Despite percentile analysis showing that infants of the 5th and 10th percentile caught up with motor skills by 18 months of age, we insist on being cautious about observing the development of these groups. Darrah et al. defined two cut-off diagnostic points for identifying infants with atypical motor development – the 10th percentile at 4 months, and the 5th percentile at 8 months (Darrah et al., 2014). The Polish AIMS percentiles are relatively similar to Canadian. Differences were found particularly in the groups of 6-<7 and 15-<16 months of age. Generally, a few significant differences are diffused, with the exception of the 75th percentile, in which occurred in six age groups. Syrengelas et al. explained the existence of some diffused differences with the sensitivity of the binomial test comparing frequencies regarding percentile analogies and not absolute numbers, and may be caused by the difference in the number of sample sizes (Polish versus Canadian) [35]. Gontijo et al. received the same results for the 75th percentile. However, the authors recognized it as not clinically meaningful [32].

Considerable strengths of our study involved the development of normative values for culturally adapted and validated tool, appropriate sample size, proving both mean scores and percentile ranks of the AIMS in the Polish sample, as well as the comparison of results with the Canadian norms.

On the other hand, we have also acknowledged its limitations. The main of them is a certain homogeneity of our sample, participants inhabited the largest city of Greater Poland, its suburbs or small towns nearly located, and were born as the first or second child in families. In previous studies, participants had also been recruited from one city or province/district [16, 30, 32, 35]. We realize that our finding refers only to term infants, and thereby research on the standardization of the AIMS in Polish preterm infants is needed. Furthermore, we also opt for studies on reference norms of the AIMS scores of its particular items and positions (prone, supine, sitting, standing).

## Conclusion

Our study provides the reference values for the Polish version of the AIMS in term population aged 0-<19 months. According to differences in the mean AIMS total scores and percentiles, the original Canadian reference norms are incongruent for Polish infants. We stand for the proceeding of the standardization of the AIMS scores in any population before using the scale in clinics and research.

## Data Availability

The datasets used and/or analysed during the current study are available from the corresponding author on reasonable request.
